# Puerarin Reverses UV-Induced Epigenetic Silencing of the Wnt/β-Catenin-KIT Axis to Mitigate Skin Fibroblast Aging

**DOI:** 10.3390/ijms27104444

**Published:** 2026-05-15

**Authors:** Shixiong Zheng, Ye Hong, Yuxuan Xiao, Aliya Yijiati, Yunying Mo, Xingyu Yu, Shihan Huang, Xiaoyu Xian, Yuanyuan Jiang, Qingzhi Wei, Xingfen Yang, Zhini He

**Affiliations:** 1 Food Safety and Health Research Center, School of Public Health, Southern Medical University, Guangzhou 510515, China; 2 Guangdong Provincial Key Laboratory of Tropical Disease Research, Guangzhou 510515, China

**Keywords:** photoaging, DNA methylation, Wnt signaling, *KIT*, puerarin, epigenetic therapy

## Abstract

Ultraviolet radiation (UVR) exposure accelerates skin aging by disrupting cellular homeostasis and inducing epigenetic changes, such as promoter hypermethylation of key regulatory genes. However, the molecular mechanisms underlying UVR-driven epigenetic silencing remain poorly understood. By integrating high-throughput DNA methylation profiling with co-regulatory network analysis, we identified *KIT* as a hub gene in a photoaging-associated methylation module. Pathway enrichment further revealed coordinated hypermethylation of the canonical Wnt/β-catenin signaling pathway, establishing the Wnt/KIT axis as a critical epigenetic-signaling nexus in UVR-induced skin fibroblast aging. In immortalized human skin fibroblasts (HSFs), UVR suppressed Wnt signaling, leading to *KIT* promoter hypermethylation, transcriptional silencing, and premature photoaging. Gain-of-function studies revealed that reversing *KIT* hypermethylation either via Wnt pathway activation or *KIT* overexpression effectively mitigated photoaging-associated phenotypes. Crucially, we found that puerarin (PUE), a natural isoflavone, reversed UVR-induced epigenetic silencing by directly interacting with β-catenin, reactivating Wnt signaling, and restoring *KIT* expression. PUE treatment preserved cellular function in UVR-damaged fibroblasts. These findings establish the Wnt/β-catenin-KIT axis as a critical epigenetic driver of skin aging and highlight puerarin as a promising therapeutic candidate for targeted anti-aging intervention.

## 1. Introduction

Photoaging refers to premature skin aging caused by chronic UVR exposure, histopathologically characterized by dermal extracellular matrix degradation (collagen fragmentation, and elastin accumulation) and fibroblast dysfunction [1]. Epidemiological evidence attributes up to 80% of visible skin aging to environmental factors, with UVR being the primary driver. Its pathogenic mechanisms include DNA damage, reactive oxygen species (ROS)-mediated oxidative stress, and dysregulated matrix metalloproteinase activity—all leading to collagen depletion [2]. UVA (~90% of terrestrial UVR) penetrates deeply into the dermis, causing persistent oxidative stress and mitochondrial dysfunction; UVB (~10%) triggers acute inflammation and cyclobutane pyrimidine dimer formation. Their combined effects underpin photoaging progression [3]. Beyond aesthetics, photoaging increases the risk of actinic keratosis and non-melanoma skin cancer, and imposes psychosocial burdens on quality of life [4,5].

Recent advances position DNA methylation as a dynamic, environmentally responsive regulator of skin aging—serving as both a mediator of UVR damage and a potential biomarker [6]. Age-associated methylation changes (global hypomethylation accompanied by locus-specific hypermethylation) are functionally consequential, as they alter the expression of genes governing cell aging and matrix homeostasis [7]. Critically, UVR induces rapid, persistent methylome remodeling in skin cells [8], suggesting epigenetic dysregulation contributes causally to photoaging.

*KIT*—a receptor tyrosine kinase—has emerged as an epigenetically regulated node in aging: hypermethylation-mediated KIT silencing occurs in age-related pathologies, and demethylation restores its protective functions [9,10,11]. Concurrently, the Wnt/β-catenin pathway maintains skin homeostasis by promoting fibroblast proliferation and *COL1A1* expression [12,13]. Wnt signaling also modulates DNA methylation—either preserving epigenetic stability in stem cells [14] or driving locus-specific hypermethylation via *DNMT1* upregulation [15]. Clinical observations link co-dysregulation of Wnt and *KIT* to skin disorders (e.g., atopic dermatitis) [16,17], and dual-pathway modulation improves pathological phenotypes [18], implying functional crosstalk.

Puerarin (PUE)—a major isoflavone from *Pueraria lobata*—exhibits multi-target anti-aging effects and a favorable safety profile [19,20]. Recent studies suggest that puerarin exerts protective effects in multiple aging models: in vivo, it alleviates D-galactose-induced systemic photoaging [21]; at the cellular level, it impedes replicative photoaging in dermal fibroblasts [22]; notably, in ultraviolet radiation (UVR)-induced photoaging models, it provides effective protection by activating the Nrf2 pathway and suppressing the MAPK pathway [23]. Moreover, puerarin can influence cellular photoaging phenotypes by regulating non-coding RNA networks and DNA methylation [24,25].

This study aimed to: (1) integrate bioinformatics and cellular validation to elucidate the Wnt/KIT epigenetic mechanism, (2) identify *KIT* hypermethylation as a key regulator of UVR-induced photoaging, and (3) demonstrate puerarin’s protective effects in reversing UVR-induced epigenetic and phenotypic changes. Our findings provide new insights into photoaging’s epigenetic mechanisms and support PUE as a potential targeted therapy.

## 2. Results

### 2.1. Identification of UVR-Induced Differentially Methylated Genes and Pathways

**Figure 1:**
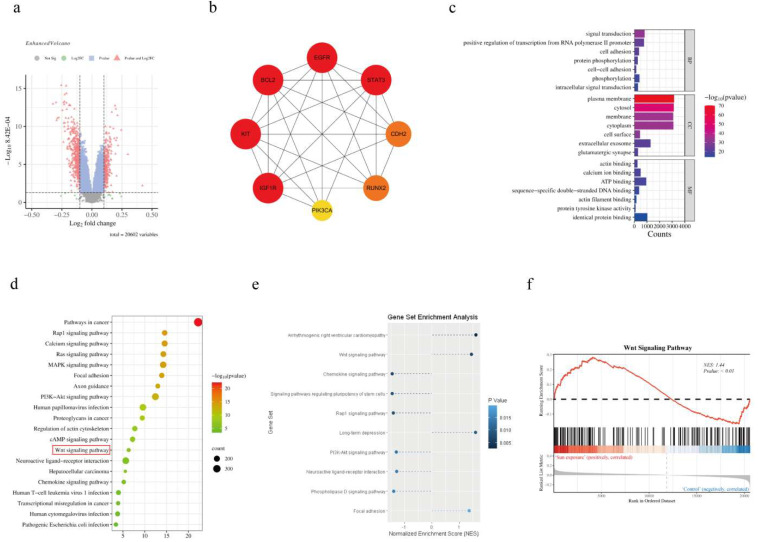
Differentially methylated genes and pathway enrichment analysis of UVR-exposed human skin. (**a**) Volcano plot of differentially methylated genes (DMGs) in sun-exposed vs. sun-protected dermal tissues (GSE51954 dataset; *n* = 20 young adults). Red triangle = probes with FDR < 0.05 and |Δβ| > 0.1; Blue square = probes with FDR < 0.05 and |Δβ| < 0.1. (**b**) Protein–protein interaction (PPI) network of DMGs (STRING, interaction score > 0.4) with hub genes highlighted in red. (**c**) Top 10 Gene Ontology (GO) biological process terms enriched in DMGs (clusterProfiler, *p* < 0.05). (**d**) KEGG pathway enrichment of DMGs, with the Wnt/β-catenin pathway marked in red. (**e**) Gene Set Enrichment Analysis (GSEA) of UVR-exposed tissues, showing enrichment of “aging-related pathways.” (**f**) GSEA plot of the Wnt/β-catenin pathway, demonstrating coordinated hypermethylation in UVR-exposed samples (*NES* = −1.8, *FDR* = 0.03).

To uncover epigenetic regulators of photoaging, we analyzed the GEO dataset GSE51954—comprising genome-wide DNA methylation profiles of sun-exposed and sun-protected dermal tissues from 20 healthy young adults (20–30 years old). We identified 676 differentially methylated genes (DMGs; FDR < 0.05, |Δβ| > 0.1), including 274 hypermethylated and 402 hypomethylated genes (Figure 1a). A protein–protein interaction (PPI) network analysis of DMGs identified eight hub genes: *EGFR*, *KIT*, *STAT3*, *BCL2*, *CDH2*, *PIK3CA*, *RUNX2*, and *IGF1R* (Figure 1b). Among these, *KIT*, *BCL2*, and *CDH2* were hypermethylated, while *EGFR*, *STAT3*, *PIK3CA*, *RUNX2*, and *IGF1R* were hypomethylated. KEGG and GSEA analyses further revealed that the canonical Wnt/β-catenin pathway was coordinately hypermethylated in UVR-exposed tissues (Figure 1c–f), suggesting its epigenetic silencing in photoaging.

### 2.2. UVR Induces HSF Photoaging and Downregulates KIT

**Figure 2:**
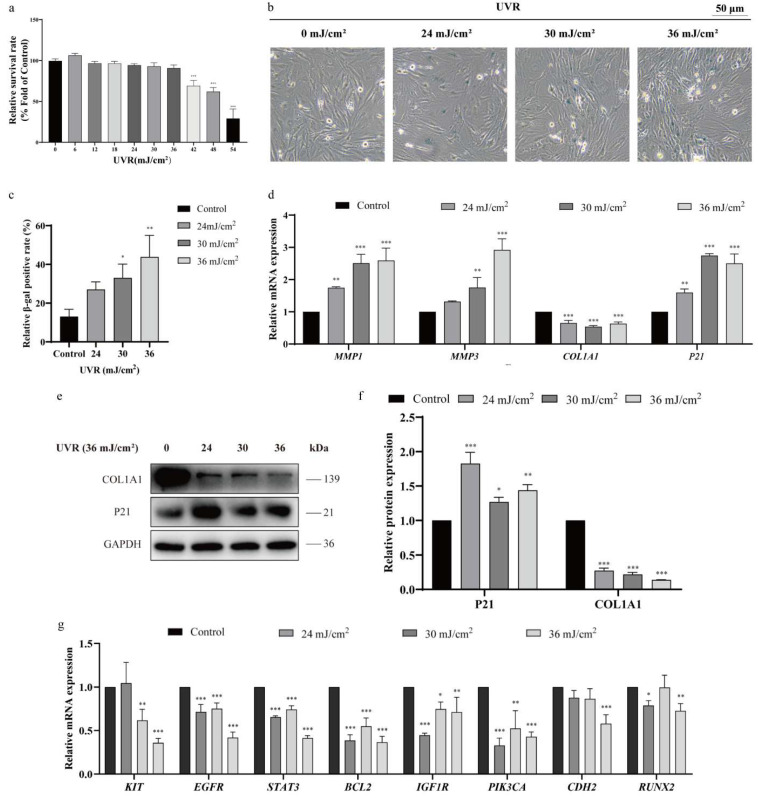
UVR dose-dependently induces photoaging and downregulates hub genes in HSFs. (**a**) MTT assay of HSFs viability after 24 h UVR exposure (6–54 mJ/cm^2^; *n* = 3 independent experiments). (**b**) Representative images of SA-β-gal staining (blue) in HSFs treated with 0, 30, or 36 mJ/cm^2^ UVR (scale bar = 50 μm). (**c**) Quantification of SA-β-gal-positive cells (*n* = 3 fields/well, ≥200 cells/field). (**d**) qRT-PCR analysis of *MMP-1*, *MMP-3*, *P21*, and *COL1A1* mRNA (*n* = 3). (**e**) Western blot of *COL1A1* and *P21* protein; *GAPDH* as loading control. (**f**) Quantification of Western blot band intensities (ImageJ; *n* = 3). (**g**) qRT-PCR of hub gene mRNA in UVR-treated HSFs (*n* = 3). Statistical significance: * *p* < 0.05, ** *p* < 0.01, and *** *p* < 0.001 vs. control (one-way ANOVA + Bonferroni test).

We first determined the non-cytotoxic UVR dose range for HSFs via MTT assay: UVR doses ≤ 36 mJ/cm^2^ did not reduce cell viability (*p* > 0.05; Figure 2a). We then tested doses of 24, 30, and 36 mJ/cm^2^ for photoaging induction. UVR at 30 mJ/cm^2^ significantly increased SA-β-gal-positive cells (*p* < 0.05; Figure 2b,c), while doses ≥ 36 mJ/cm^2^ elicited the most pronounced senescent phenotype: dose-dependent upregulation of *MMP-1*, *MMP-3*, and *P21* (photoaging marker) and downregulation of *COL1A1* (collagen synthesis marker; Figure 2d–f, *p* < 0.05). Thus, 36 mJ/cm^2^ was selected as the photoaging modeling dose.

To validate bioinformatic findings, we measured hub gene mRNA expression via qRT-PCR. All eight hub genes were generally downregulated by UVR (*p* < 0.05; Figure 2g). However, we observed non-dose-dependent expression patterns for some hub genes (e.g., *EGFR*, *STAT3*, and *PIK3CA*) as UVR dose increased. This may reflect the complexity of UVR-induced cellular stress: at low UVR doses, cells might activate compensatory mechanisms (antioxidant or anti-apoptotic pathways) that temporarily stabilize gene expression, while higher doses overwhelm these defenses, leading to more pronounced downregulation. Notably, *KIT*, *BCL2*, and *CDH2* (hypermethylated in GSE51954) showed a negative correlation between DNA methylation and mRNA expression, consistent with epigenetic silencing.

### 2.3. UVR Suppresses KIT Expression via Promoter Hypermethylation

**Figure 3:**
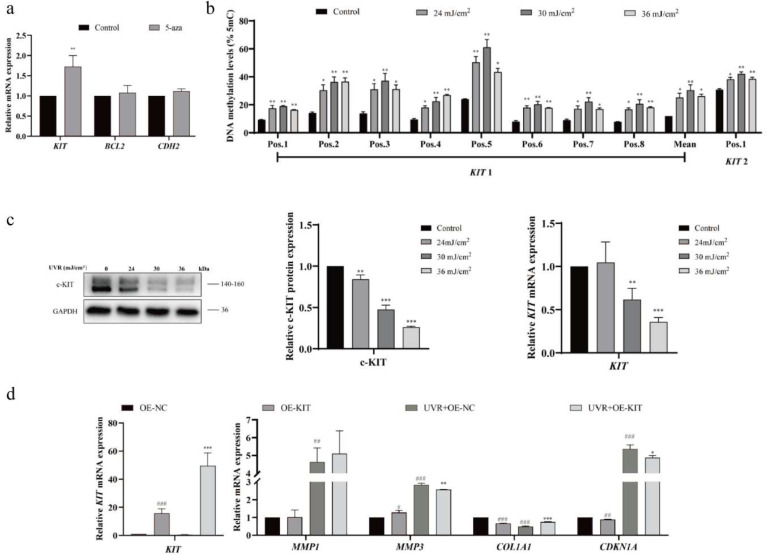
UVR suppresses *KIT* expression via promoter hypermethylation. (**a**) qRT-PCR of *KIT*, *BCL2*, and *CDH2* mRNA in HSFs treated with 5-azacytidine (5-aza; 10 μM, 72 h; *n* = 3). (**b**) Pyrosequencing quantification of *KIT* DNA methylation (*KIT-1*: −145 to −108 bp relative to TSS; *KIT-2*: 3115 to 3126 bp) in HSFs treated with 0, 24, 30, 36 mJ/cm^2^ UVR (*n* = 3). (**c**) Western blot (left) and qRT-PCR (right) of c-*KIT* protein and *KIT* mRNA in UVR-treated HSFs (*n* = 3). (**d**) qRT-PCR of *MMP-1*, *MMP3*, *COL1A1*, and *CDKN1A* mRNA in HSFs transfected with KIT overexpression plasmid (OE-*KIT*) or empty vector (OE-NC) + 36 mJ/cm^2^ UVR (*n* = 3). Statistical significance: * *p* < 0.05, ** *p* < 0.01, and *** *p* < 0.001 vs. OE-NC group; ^#^ *p* < 0.05, ^##^ *p* < 0.01, ^###^ *p* < 0.001 vs. UVR+OE-NC group (*t*-test or one-way ANOVA + Bonferroni test).

To confirm epigenetic regulation of *KIT*, we treated HSFs with the DNA methyltransferase inhibitor 5-azacytidine (5-aza). 5-aza significantly upregulated *KIT* mRNA (*p* < 0.01) but did not affect *BCL2* or *CDH2* (Figure 3a), indicating *KIT* is specifically regulated by DNA methylation.

Pyrosequencing revealed UVR-induced hypermethylation at two target regions of *KIT*: *KIT-1* (−145 to −108 bp relative to TSS) increased from 11.92% to 27.15%, and *KIT-2* (3115 to 3126 bp) from 31.28% to 39.30% (*p* < 0.05; Figure 3b). Concomitantly, *KIT* mRNA and c-*KIT* protein were dose-dependently downregulated by UVR (*p* < 0.05; Figure 3c), linking promoter hypermethylation to transcriptional silencing.

To test *KIT*’s functional role, we overexpressed *KIT* in HSFs (≈50-fold increase in mRNA; *p* < 0.001; Figure 3d). Furthermore, KIT overexpression rescued UVR-induced photoaging: it downregulated *MMP-1* and *P21* and upregulated *COL1A1* at both mRNA and protein levels (*p* < 0.05; Figure 3d), restoring extracellular matrix homeostasis and mitigating cell cycle arrest.

### 2.4. Wnt/β-Catenin Pathway Modulates Photoaging by Regulating KIT Methylation

**Figure 4:**
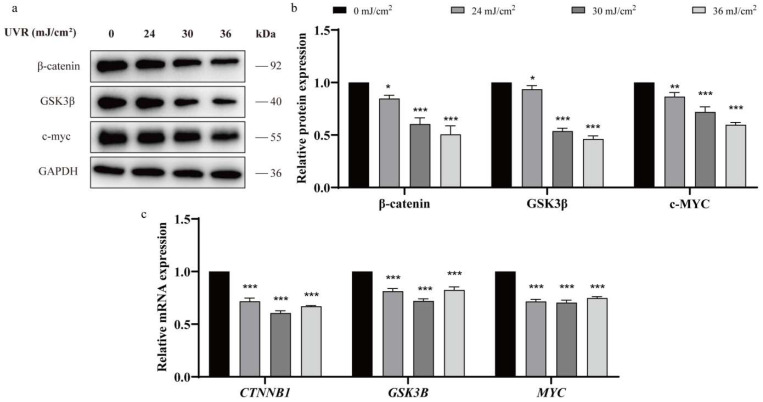
UVR dose-dependently suppresses Wnt/β-catenin signaling in HSFs. (**a**) Western blot of Wnt pathway proteins (β-catenin, GSK3β, c-*MYC*) in HSFs treated with 0–36 mJ/cm^2^ UVR; *GAPDH* as loading control. (**b**) Quantification of Western blot band intensities (ImageJ; *n* = 3). (**c**) qRT-PCR of *CTNNB1*, *GSK3B*, and *MYC* mRNA in UVR-treated HSFs (*n* = 3). Statistical significance: * *p* < 0.05, ** *p* < 0.01, and *** *p* < 0.001 vs. 0 mJ/cm^2^ (one-way ANOVA + Bonferroni test).

UVR (36 mJ/cm^2^) dose-dependently downregulated key Wnt pathway components: *CTNNB1* (β-catenin), *GSK3B*, and *MYC* (*p* < 0.05; Figure 4a–c), indicating Wnt signaling suppression in photoaging.

**Figure 5:**
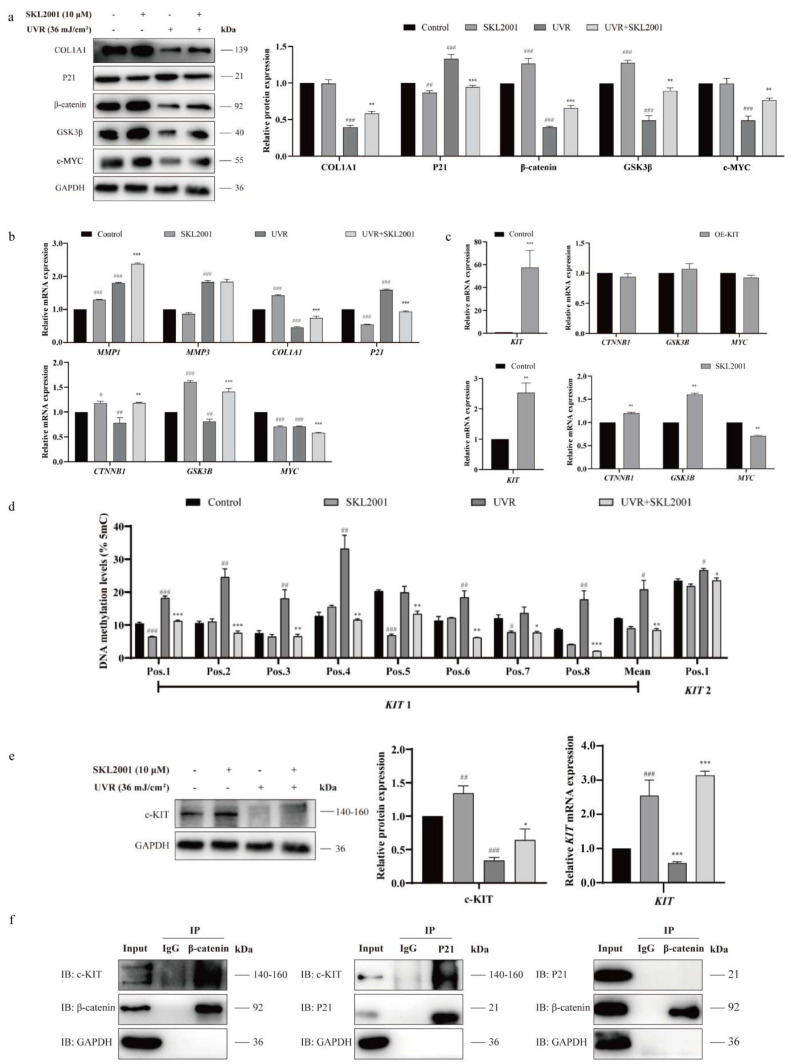
Wnt signaling regulates *KIT* methylation and photoaging. (**a**) Western blot (left) and quantification of Western blot band intensities (right) of *COL1A1*, *P21*, β-catenin, etc. In HSFs treated with 36 mJ/cm^2^ UVR ± SKL2001 (10 μM, Wnt agonist; *n* = 3). (**b**) qRT-PCR of photoaging-related genes and key Wnt pathway molecules in the same groups (*n* = 3). (**c**) qRT-PCR of *KIT* and Wnt signaling components’ mRNA in HSFs treated with SKL2001 or OE-*KIT* (*n* = 3). (**d**) Pyrosequencing of *KIT* DNA methylation in HSFs treated with UVR ± SKL2001 (*n* = 3). (**e**) Western blot of c-*KIT* in the same groups (*n* = 3). (**f**) Co-immunoprecipitation (Co-IP) of c-*KIT*, β-catenin, and *P21* in HSFs (*n* = 2 independent experiments). Statistical significance: * *p* < 0.05, ** *p* < 0.01, *** *p* < 0.001 vs. control; ^#^ *p* < 0.05, ^##^ *p* < 0.01, ^###^ *p* < 0.001 vs. UVR alone (*t*-test).

We then activated Wnt signaling with SKL2001 (10 μM), a specific agonist. SKL2001 reversed UVR-induced Wnt inhibition (*p* < 0.05; Figure 5a), upregulated *COL1A1*, and downregulated *P21* (*p* < 0.05; Figure 5b), preserving collagen synthesis and alleviating aging.

To define the regulatory hierarchy: *KIT* overexpression did not alter Wnt pathway activity, but SKL2001 upregulated *KIT* mRNA and protein (*p* < 0.05; Figure 5c), placing *KIT* downstream of Wnt. Critically, SKL2001 reversed UVR-induced *KIT* promoter hypermethylation (*p* < 0.05; Figure 5d,e), linking Wnt activation to epigenetic reactivation of *KIT*. Co-immunoprecipitation (Co-IP) showed c-*KIT* forms a complex with β-catenin and *P21*, but β-catenin does not directly interact with *P21* (Figure 5f), suggesting Wnt/β-catenin may regulate aging via c-*KIT*-*P21* interactions.

### 2.5. Puerarin Delays HSF Aging via the Wnt/KIT Axis

**Figure 6:**
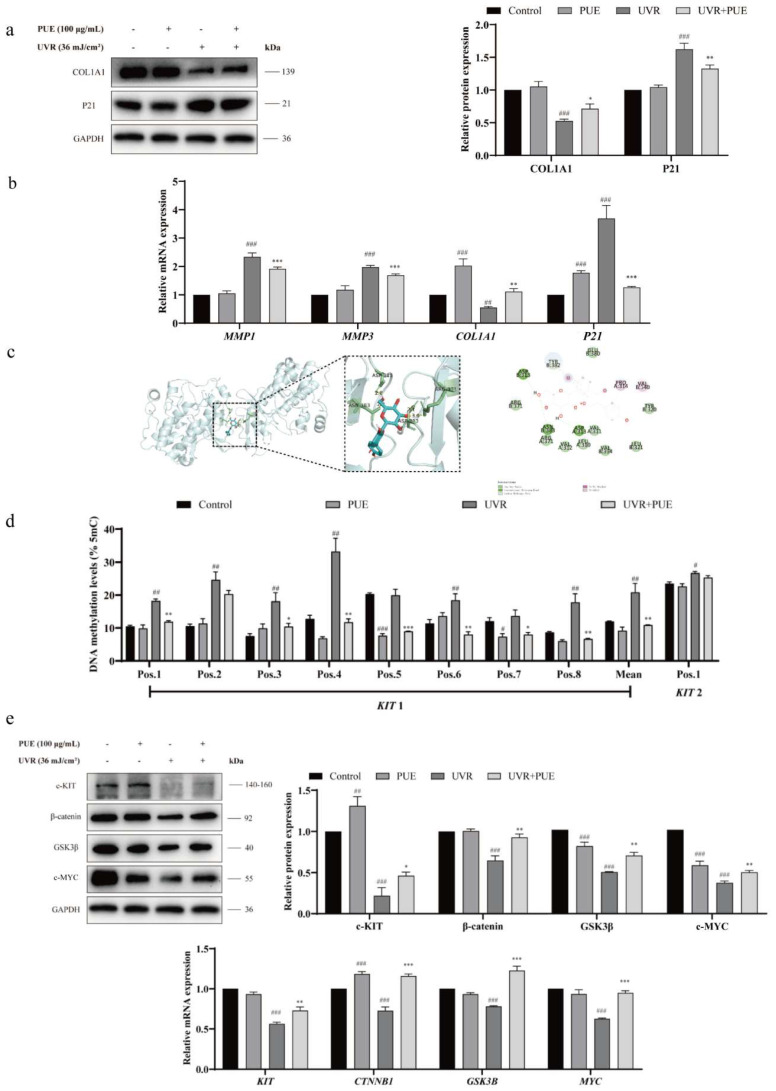
Puerarin mitigates UVR-induced photoaging via the Wnt/KIT axis. (**a**) Western blot (left) and quantification of Western blot band intensities (right) of *COL1A1* and *P21* in HSFs treated with 36 mJ/cm^2^ UVR ± puerarin (PUE; 100 μM, 24 h; *n* = 3). (**b**) qRT-PCR of photoaging-related genes in the same groups (*n* = 3). (**c**) Molecular docking of PUE with β-catenin (PDB: 3BCT): binding pose (left) and interaction details (right; ΔG = −8.5 kcal/mol). (**d**) Pyrosequencing of *KIT* DNA methylation in HSFs treated with UVR ± PUE (*n* = 3). (**e**) Western blot of c-*KIT*, β-catenin, *GSK3β*, and c-*MYC* in the same groups (*n* = 3). Statistical significance: * *p* < 0.05, ** *p* < 0.01, *** *p* < 0.001 vs. Control; ^#^ *p* < 0.05, ^##^ *p* < 0.01, ^###^ *p* < 0.001 vs. UVR alone (*t*-test or one-way ANOVA + Bonferroni test).

In UVR-induced senescent HSFs, PUE treatment significantly mitigated photoaging phenotypes, as evidenced by the downregulation of *MMP-1* and *P21* and the upregulation of *COL1A1* (Figure 6a,b). Molecular docking predicted a high-affinity interaction between PUE and β-catenin (ΔG = −8.5 kcal/mol; Figure 6c), suggesting its potential to directly modulate the Wnt pathway. Consistent with this, PUE reversed UVR-induced *KIT* promoter hypermethylation (*p* < 0.05; Figure 6d), concomitant with the restoration of c-*KIT* protein expression. Furthermore, PUE co-treatment counteracted the UVR-mediated suppression of β-catenin and showed a tendency to restore the expression of its downstream target c-*MYC*, although the effect on *GSK3β* was less pronounced (Figure 6e). Notably, PUE alone did not significantly alter the levels of these axis components, indicating that its primary role is to antagonize UVR-induced damage rather than to constitutively activate the pathway. Collectively, these data suggest that PUE, likely via binding to β-catenin, mitigates UVR-induced epigenetic silencing of *KIT*, leading to the restoration of *KIT* expression and the subsequent reactivation of the Wnt/β-catenin signaling node. This molecular reprogramming ultimately underlies the observed attenuation of HSF photoaging.

## 3. Discussion

Skin, the body’s largest organ, is uniquely vulnerable to environmental stressors—with UVR being the primary extrinsic driver of premature aging (photoaging) [2]. Unlike intrinsic aging, photoaging is characterized by accelerated dermal extracellular matrix degradation, fibroblast photoaging, and paracrine-mediated “bystander photoaging” that propagates dysfunction to neighboring cells [26,27]. While UVR supports physiological processes like vitamin D synthesis [28], its classification as a Group 1 carcinogen by the IARC underscores its pathological impact [29]. For instance, in vivo studies using a hair follicle photoaging model have shown that UVR, particularly UVB, can upregulate the expression of Wnt pathway components [30]. However, findings from other in vitro systems have demonstrated that UVB irradiation suppresses Wnt/β-catenin activity in dermal fibroblasts and reduces β-catenin expression in keratinocytes [31,32], which is consistent with our observations. Using a solar-simulated UVR model (290–400 nm, Solar Light 601), we define a novel epigenetic circuit linking UVR to photoaging: UVR inhibits Wnt/β-catenin signaling, driving *KIT* promoter hypermethylation, transcriptional silencing, and subsequent fibroblast photoaging. This work identifies the Wnt/KIT axis as a critical environment-epigenetics nexus in dermal aging.

Epigenetic modifications—particularly DNA methylation—are now recognized as reversible mediators of aging, bridging environmental cues to persistent phenotypic changes [33,34]. Lifestyle interventions (e.g., smoking cessation, healthy diets) can correct aberrant methylation and reduce age-related disease risk [35,36], but targeted epigenetic therapies for photoaging remain underdeveloped. Unlike prior studies focusing on global methylome shifts [8], we demonstrate that physiologically relevant UVR induces gene-specific hypermethylation (e.g., *KIT*) in fibroblasts, suggesting chronic low-dose UV exposure progressively dysregulates epigenetic control via discrete signaling nodes. Our functional validation confirms that reversing *KIT* hypermethylation (via 5-azacytidine or Wnt activation) restores *KIT* expression and ameliorates aging phenotypes—consistent with reports of *KIT* epigenetic silencing in melanoma [37]. These findings align with *KIT*’s established role in skin homeostasis: it promotes DNA repair [38], recruits stem cells for tissue regeneration [39], and modulates cell adhesion in pathological repair (e.g., corneal epithelial healing [40], diabetic wounds [41]). Together, our data position *KIT* as a key epigenetic “gatekeeper” of dermal youth.

This study reveals a regulatory hierarchy between suppressed Wnt/β-catenin signaling and *KIT* promoter hypermethylation in the context of UVR-induced photoaging. UVR exposure downregulates Wnt pathway activity (evidenced by reduced expression of *CTNNB1*, *GSK3B*, and *MYC*), an event closely associated with *KIT* promoter hypermethylation and subsequent transcriptional silencing. The functional relevance of this association is supported by two lines of evidence: pharmacological activation of Wnt signaling with SKL2001 reversed UVR-induced *KIT* hypermethylation and restored *KIT* expression, whereas inhibition of DNA methylation with 5-azacytidine specifically upregulated *KIT*, confirming that its expression is under direct epigenetic control.

Although the molecular link between Wnt suppression and DNMT recruitment to the *KIT* locus remains to be fully elucidated, existing studies provide a plausible mechanistic framework. UVR acts as a key environmental driver of epigenetic reprogramming in the skin, capable of inducing promoter-specific hypermethylation of genes such as *KIT* through mechanisms involving oxidative stress, alterations in histone modifications (e.g., H3K9me3), and modulation of epigenetic regulatory enzymes [42,43]. Our work establishes the Wnt/KIT axis as a critical and targetable epigenetic interface in dermal fibroblasts. By proposing epigenetic reprogramming as a dynamic mechanism underlying their coordinated dysregulation, this model extends previous observations of Wnt/KIT co-dysregulation in skin disorders [16,17,44], aligning with the conserved role of Wnt signaling in guiding cell fate through epigenetic modulation in other systems [45,46].

We further identify PUE as a natural modulator of the Wnt/KIT axis. PUE reversed UVR-induced *KIT* hypermethylation, restored collagen homeostasis, and mitigated photoaging—consistent with its broad epigenetic activity (e.g., regulating ERα/NF-κB pathways [47], histone methylation in endothelial cells [25]). Furthermore, PUE exerts its protective effects through multiple complementary mechanisms. Beyond the epigenetic regulation emphasized here, PUE has been shown to directly downregulate the mRNA expression of key stress-response kinases in the MAPK pathway (*JNK*, *ERK*, and *p38*) in UVA-irradiated dermal fibroblasts, thereby reducing *MMP-1* overexpression and alleviating extracellular matrix degradation [23]. Moreover, PUE’s action extends to modulating fundamental cellular processes such as stem cell proliferation, differentiation, and apoptosis, which are vital for tissue repair and regeneration. This multi-target capacity underscores its potential to intervene in the aging process at both the molecular and cellular levels [48]. These data provide mechanistic support for PUE’s traditional use in anti-aging therapies and position it as a lead compound for photoprotection: unlike synthetic epigenetic drugs (e.g., 5-aza), PUE exhibits a favorable safety profile and potential for topical delivery [20].

## 4. Materials and Methods

### 4.1. Cell Culture

Immortalized human skin fibroblasts (HSFs) were purchased from Sunncell Biotechnology (Wuhan, China; derived from male foreskin tissue, passage 3–6; Cat. No. SNL-500). Cells were cultured in Dulbecco’s Modified Eagle Medium (DMEM; Gibco, Grand Island, NY, USA), supplemented with 10% fetal bovine serum (FBS; Excell, Shanghai, China), 1% penicillin-streptomycin (Gibco), 1 mM sodium pyruvate, and 2 mM L-glutamine. Cells were maintained at 37 °C in a humidified incubator with 5% CO_2_ (Thermo Scientific, Waltham, MA, USA) and passaged when reaching 80% confluence to avoid replicative photoaging.

### 4.2. Bioinformatic Analysis

To identify key epigenetic regulators of UV-induced skin photoaging, we analyzed the GEO dataset GSE51954 (accession number: GSE51954; accessed on 29 May 2023), which contains genome-wide DNA methylation profiles of sun-exposed and sun-protected dermal tissues from 20 healthy young adults (20–30 years old). Differential methylation analysis was performed using the “limma” R package (v3.52.2) with the following thresholds: false discovery rate (FDR) < 0.05 and absolute methylation difference (|Δβ|) > 0.1. Differentially methylated genes (DMGs) were subjected to Gene Ontology (GO) biological process and Kyoto Encyclopedia of Genes and Genomes (KEGG) pathway enrichment analyses using clusterProfiler (v4.4.4; *p* < 0.05, *q* < 0.2). A protein–protein interaction (PPI) network was constructed via STRING (v11.5; interaction score > 0.4) and visualized in Cytoscape (v3.9.1). Hub genes were identified using the Maximal Clique Centrality (MCC) algorithm in CytoHubba. Gene Set Enrichment Analysis (GSEA; v4.3.2) was conducted to detect coordinately regulated pathways (normalized enrichment score [NES] > 1.5, FDR < 0.25).

### 4.3. Cell Viability Assay

The optimal UVR and PUE doses were determined via MTT assay. HSFs were seeded in 96-well plates (8 × 10^3^ cells/well) and incubated for 24 h. Cells were treated with either UVR (290–400 nm, SolarLight 601; SolarLight, Glenside, PA, USA) at 6–54 mJ/cm^2^ alone or PUE (purity ≥ 98%; Solarbio, Beijing, China) at 0–1000 μg/mL for 24 h. MTT solution (0.5 mg/mL, Sigma-Aldrich, St. Louis, MO, USA) was added for 4 h. Then, the supernatant was removed and 150 μL dimethyl sulfoxide (DMSO, Sigma-Aldrich) was added to dissolve the formazan crystals. Absorbance was measured at 490 nm using a microplate spectrophotometer (Epoch-BioTek, Beijing, China). All experiments were performed in triplicate.

### 4.4. UVR Irradiation and Experimental Grouping

Based on the maximum non-toxic dose determined in the MTT assay and relevant literature, HSFs were divided into groups and dosed as follows: Blank Control group (no UV irradiation), PUE Treatment group (100 μg/mL PUE), UVR group (36 mJ/cm^2^ UVR), and UVR+PUE group (36 mJ/cm^2^ UVR + 100 μg/mL PUE). In the UVR+PUE group, cells were first irradiated with UVR, followed by PUE treatment.

The UV irradiation protocol was as follows: Culture dishes were placed under the UVR source and completely covered, with a distance of 3.1 cm between the source and the cell surface, and irradiation was performed at room temperature. To ensure the reproducibility of the experimental results, the UV intensity measurement conditions were identical to the experimental setup: the detector was placed 3.1 cm from the source and completely covered.

### 4.5. Quantitative Real-Time PCR (qRT-PCR)

Total RNA was extracted with TRIzol (Invitrogen, Waltham, MA, USA) per the manufacturer’s protocol. cDNA was synthesized using the Evo M-MLV Reverse Transcriptase Kit (Accurate Biotechnology, Guangzhou, China). qRT-PCR was performed on a StepOnePlus Real-Time PCR System (Applied Biosystems, Foster City, CA, USA) with SYBR Green Master Mix (Accurate Biotechnology). Primers targeting *MMP-1*, *MMP-3*, *P21*, *COL1A1*, and *GAPDH* (internal control) are listed in Table S1. Relative gene expression was calculated using 2^−ΔΔCt^.

### 4.6. Western Blot Analysis

Proteins were extracted with RIPA lysis buffer (Beyotime Biotechnology, Shanghai, China) supplemented with 1 mM phenylmethylsulfonyl fluoride (PMSF; Solarbio) and 1× phosphatase inhibitor cocktail (Roche, Rotkreuz, Switzerland). Protein concentration was quantified via BCA assay (Solarbio). Equal amounts of protein (20 μg/lane) were separated by 10% SDS-PAGE and transferred to polyvinylidene fluoride (PVDF) membranes (Millipore, Billerica, MA, USA). Membranes were blocked with 5% non-fat milk in TBST (10 mM Tris-HCl, 150 mM NaCl, 0.1% Tween-20) for 1 h at room temperature, then incubated overnight at 4 °C with primary antibodies: anti-c-*KIT* (1:1000; Proteintech, Chicago, IL, USA, #18696-1-AP), anti-β-catenin (1:5000; Abmart, Wuhan, China, #M24002F), anti-*COL1A1* (1:2000; Abcam, Cambridge, UK, ab34710), anti-*P21* (1:1000; Proteintech, #10355-1-AP), and anti-*GAPDH* (1:5000; Proteintech, #60004-1-Ig). After washing, membranes were incubated with HRP-conjugated secondary antibodies (1:2000; Cell Signaling Technology [CST], Danvers, MA, USA, #7074 [rabbit] or #7076 [mouse]) for 1 h at room temperature. Signals were detected using ECL substrate (Beyotime) and imaged with a Tanon 5200 chemiluminescence system (Tanon Technology, Shanghai, China). Band intensities were quantified via ImageJ (v1.8.0).

### 4.7. Photoaging-Associated β-Galactosidase (SA-β-Gal) Staining

Senescent cells exhibit high activity of β-galactosidase. Using in situ staining with X-Gal as a substrate, an insoluble dark blue product is generated under the catalysis of β-galactosidase. Cells showing this dark blue coloration, which can be observed and counted under an optical microscope, serve as morphological evidence of cellular photoaging. Meanwhile, the proportion of positive cells is calculated for semi-quantitative assessment of the degree of photoaging.

HSFs were seeded in 6-well plates (2 × 10^5^ cells/well) and treated with UVR or PUE. SA-β-gal activity was assessed using the Photoaging-Associated β-Galactosidase Staining Kit (C0602, Beyotime) according to the manufacturer’s protocol (incubation at 37 °C without CO_2_ for 16 h). Stained cells were imaged under a light microscope (Olympus, Tokyo, Japan), and SA-β-gal-positive cells were counted in three random fields per well (≥200 cells/field). The aging rate was calculated as (positive cells/total cells) × 100%. All experiments were performed in triplicate.

### 4.8. Pyrosequencing

Genomic DNA was extracted using the TIANamp Genomic DNA Kit (Tiangen Biotech, Beijing, China) and bisulfite-converted with the EpiTect Fast DNA Bisulfite Kit (Qiagen, Hilden, Germany). Target regions of the *KIT* promoter were amplified using PyroMark PCR Master Mix (Qiagen) and biotinylated primers designed via PyroMark Assay Design 2.0 (Qiagen; Table S2). Two regions were analyzed: *KIT-1* (positions −145 to −108 bp relative to the transcription start site [TSS], CpG island-predicted) and *KIT-2* (positions 3115 to 3126 bp relative to TSS, DMG-derived). Pyrosequencing was performed on the PyroMark Q24 platform (Qiagen), and methylation levels were quantified using PyroMark Q24 Software (v2.0.6).

### 4.9. Plasmid Construction and Cell Transfection

To investigate the functional role of the *KIT* gene in UVR-induced photoaging of HSFs, this study employed a gene overexpression technique. Specifically, the full-length human *KIT* coding sequence was cloned into the pcDNA3.1(+) vector (Invitrogen) to generate the *KIT* overexpression plasmid. The recombinant plasmid was transformed into *E. coli* DH5α cells (Vazyme, Nanjing, China) and cultured in LB medium with ampicillin (100 μg/mL) at 37 °C, 200 rpm for 16 h. Plasmid DNA was isolated using the FastPure EndoFree Plasmid Mini Kit (Vazyme). HSFs at 80% confluence were transfected with 2 μg plasmid using Lipofectamine 3000 (Invitrogen) in serum-free Opti-MEM medium (Gibco). Transfection efficiency was evaluated via qRT-PCR (≥2-fold increase in *KIT* mRNA). By artificially upregulating *KIT* expression in HSFs, we simulate the reversal of *KIT* suppression observed during the photoaging process, thereby directly validating the impact of altered *KIT* expression levels on the cellular photoaging phenotype.

### 4.10. Co-Immunoprecipitation

Co-immunoprecipitation enables the specific enrichment of a target protein and its interacting proteins or complexes from a sample by using a specific antibody. To validate the direct protein—protein interactions among *KIT*, β-catenin, and *P21*, co-immunoprecipitation assays were performed in this study. HSFs were harvested, and total protein was extracted using RIPA lysis buffer. The lysate was incubated with a primary antibody overnight at 4 °C. Protein A/G agarose beads were then added to capture the immune complexes, followed by washing with PBS. Bound proteins were eluted by boiling in 2 × Laemmli loading buffer. The eluates were analyzed by Western blotting using corresponding primary antibodies to confirm specific co-precipitation.

### 4.11. Statistical Analysis

Data are presented as mean ± standard deviation (SD) of three independent experiments. Statistical analyses were performed using IBM SPSS Statistics 26 (IBM, Armonk, NY, USA). Two-group comparisons were conducted via independent samples *t*-tests; multiple-group comparisons used one-way ANOVA with Bonferroni post hoc tests. Significance was defined as * *p* < 0.05, ** *p* < 0.01, and *** *p* < 0.001.

## 5. Limitations and Future Directions

This study has several limitations: (1) We focused on fibroblasts in monoculture, but photoaging involves complex crosstalk between fibroblasts, keratinocytes, and immune cells—future work using 3D skin equivalents or in vivo models will clarify tissue-specific regulation of the Wnt/KIT axis. (2) Our acute UVR model (24–36 mJ/cm^2^) does not recapitulate chronic low-dose exposure in natural settings; a long-term irradiation model will better reflect physiological photoaging progression. (3) While we validate *KIT* methylation as a functional target, clinical correlation between *KIT* methylation and skin aging parameters (e.g., elasticity, collagen density) remains untested. (4) It is premature to conclude that the Wnt signaling pathway directly regulates *KIT* methylation based on the current data. Future studies involving DNMT participation or ChIP assays are required to establish a direct mechanistic link. (5) The current bioinformatic analysis relies on a single dataset, which may limit generalizability. Future studies should validate these findings using independent datasets to enhance robustness and clinical relevance.

Future research should prioritize: (1) Translational studies linking *KIT* promoter methylation to clinical photoaging phenotypes (e.g., via non-invasive skin biopsies and elasticity measurements); (2) Optimizing PUE delivery systems (e.g., nanocarriers) to enhance cutaneous bioavailability; (3) Clarifying the mechanism by which the Wnt signaling pathway directly regulates *KIT* methylation, subsequent studies should incorporate DNMT participation or ChIP assays; (4) Investigating whether the Wnt/KIT axis interacts with other epigenetic modifiers (e.g., histone deacetylases) to regulate photoaging.

## 6. Conclusions

Our work reveals a novel epigenetic association in photoaging: UVR-induced suppression of Wnt/β-catenin signaling is accompanied by *KIT* promoter hypermethylation and subsequent fibroblast photoaging. We propose that the Wnt/KIT axis is a target for epigenetic intervention and position PUE as a natural modulator that directly activates Wnt/β-catenin signaling, thereby reversing *KIT* hypermethylation and exerting anti-photoaging effects. These findings extend the understanding of photoaging from descriptive phenomenology toward a testable molecular framework, highlighting epigenetic modulation of the Wnt/KIT axis as a potential strategy for developing targeted anti-aging therapies aimed at the upstream regulatory events of skin aging.

## Data Availability

The original contributions presented in this study are included in the article/Supplementary Materials. Further inquiries can be directed to the corresponding author.

## Supplementary Materials

The following supporting information can be downloaded at: https://www.mdpi.com/article/10.3390/ijms27104444/s1.
