# Supplementary material for: Puerarin Reverses UV-Induced Epigenetic Silencing of the Wnt/β-Catenin-KIT Axis to Mitigate Skin Fibroblast Aging
Source: Int J Mol Sci. 2026 May 15;27(10):4444. doi: 10.3390/ijms27104444 (PMC13208025; doi:10.3390/ijms27104444)
Supplement: Supplementary file 1 [file ijms-27-04444-s001.zip › ijms-4275049-supplementary.pdf]

Table S1. Primers for Real Time-PCR

Gene	Forward	Reverse
MMP-1	AAAATTACACGCCAGATTTGCC	GGTGTGACATTACTCCAGAGTTG
MMP-3	AGTCTTCCAATCCTACTGTTGCT	TCCCCGTCACCTCCAATCC
TP53	CAGCACATGACGGAGGTTGT	TCATCCAAATACTCCACACGC
CDKN1A	TGTCCGTCAGAACCCATGC	AAAGTCGAAGTTCCATCGCTC
COL1A1	GAGGGCCAAGACGAAGACATC	CAGATCACGTCATCGCACAAC
CTNNB1	AAAGCGGCTGTTAGTCACTGG	CGAGTCATTGCATACTGTCCAT
GSK3B	GGCAGCATGAAAGTTAGCAGA	GGCGACCAGTTCTCCTGAATC
MYC	GGCTCCTGGCAAAAGGTCA	CTGCGTAGTTGTGCTGATGT
KIT	CGTTCTGCTCCTACTGCTTCG	CCCACGCGGACTATTAAGTCT

Table S2. Primers for *KIT* DNA methylation measurement

	Forward	Reverse	Sequencing
KIT 1	ATTTTAGGGGTGGAAAGGTG	Bio-TCCTCCCAACCCCCTCCCTCTA	AAAGAGTAGGGGTTAG
KIT 2	TTTGTTTGGAGGGTAAATTTAGTGT	Bio-AACATCATATAAACCAAAAAAAACATATCT	TTAATAAGTAAAGAGTAGTTGTAA
